# Phytochemistry and Pharmacological Activities of *Wolfiporia cocos* (F.A. Wolf) Ryvarden & Gilb

**DOI:** 10.3389/fphar.2020.505249

**Published:** 2020-09-15

**Authors:** Anzheng Nie, Yanhui Chao, Xiaochuan Zhang, Wenrui Jia, Zheng Zhou, Chunsheng Zhu

**Affiliations:** ^1^Department of Chinese Medicine, The First Affiliated Hospital of Zhengzhou University, Zhengzhou, China; ^2^Department of Pharmacy, The First Affiliated Hospital of Zhengzhou University, Zhengzhou, China

**Keywords:** *Wolfiporia cocos* (F.A. Wolf) Ryvarden & Gilb., phytochemistry, traditional uses, pharmacology, anti-tumor

## Abstract

*Poria cocos* is the dried sclerotium of *Wolfiporia cocos* (F.A. Wolf) Ryvarden & Gilb., which was the current accepted name and was formerly known as *Macrohyporia cocos* (Schwein.) I. Johans. & Ryvarden, *Pachyma cocos* (Schwein.) Fr., *Poria cocos* F.A. Wolf and *Sclerotium cocos* Schwein. It is one of the most important crude drugs in traditional Chinese medicine, with a wide range of applications in ameliorating phlegm and edema, relieving nephrosis and chronic gastritis and improving uneasiness of minds. Its extensive pharmacological effects have attracted considerable attention in recent years. However, there is no systematic review focusing on the chemical compounds and pharmacological activities of *Poria cocos*. Therefore, this review aimed to provide the latest information on the chemical compounds and pharmacological effects of *Poria cocos*, exploring the therapeutic potential of these compounds. We obtained the information of *Poria cocos* from electronic databases such as SCI finder, PubMed, Web of Science, CNKI, WanFang DATA and Google Scholar. Up to now, two main active ingredients, triterpenes and polysaccharides of *Poria cocos*, have been identified from *Poria cocos*. It has been reported that they have pharmacological effects on anti-tumor, anti-bacterial, anti-oxidant, anti-inflammatory, immunomodulation, and liver and kidney protection. The review summarizes the phytochemistry and pharmacological properties of *Poria cocos*, which suggest that researchers should focus on the development of new drugs about *Poria cocos* to make them exert greater therapeutic potential.

## Introduction

**Figure 1:**
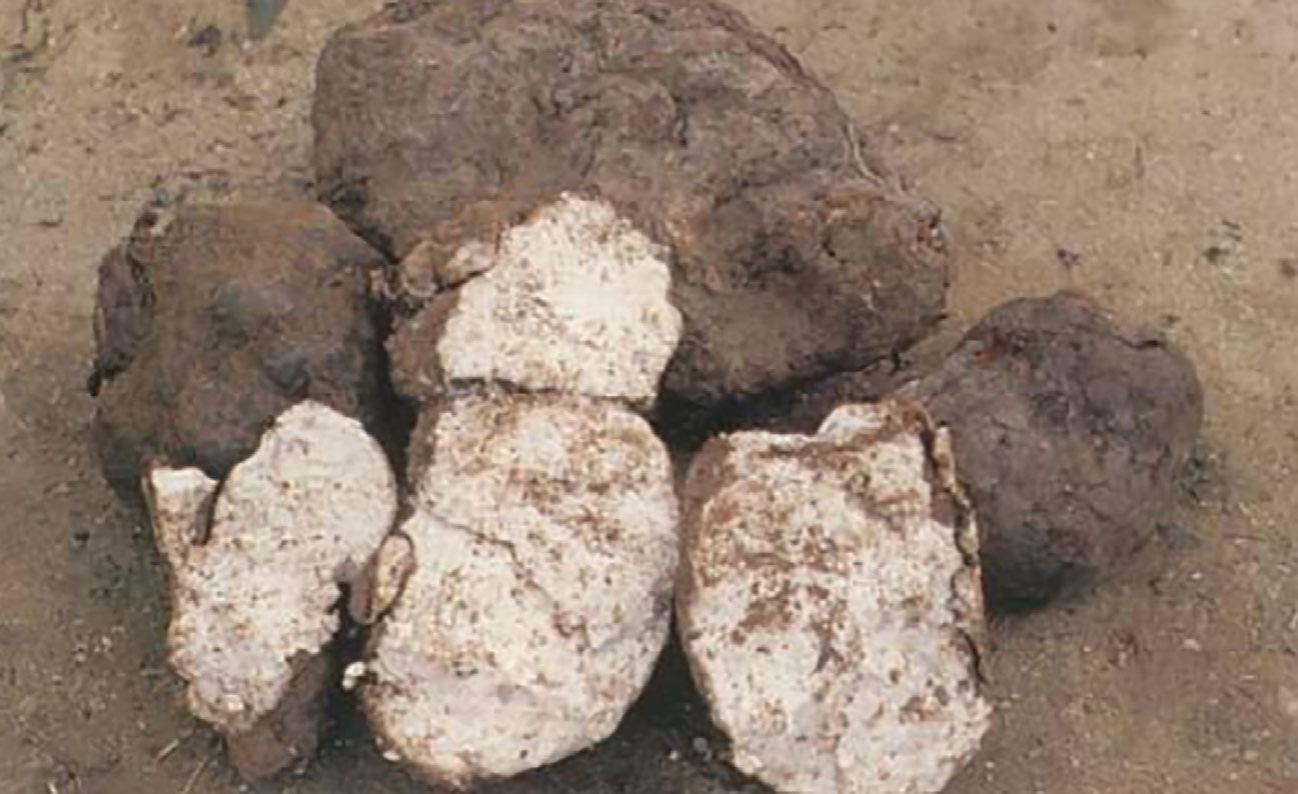
The fruiting body of Mushroom *Poria cocos. Poria cocos* has been used as famous traditional Chinese medicine known as “Fuling” in Chinese for over 2000 of years.

*Poria cocos* is the dried sclerotia of Wolfiporia cocos (F.A. Wolf) Ryvarden & Gilb., which is also referred to as “Fuling” in China (Yuan et al., 2018; Royal Botanical Gardens at Kew, 2020; Wang et al., 2020). It is a health-care edible medicinal mushroom belonging to family Polyporaceae and is firstly recorded in an ancient Chinese medical masterpiece “Sheng Nong’s herbal classic” that has been used as famous traditional Chinese medicine for over 2,000 years (Li X. et al., 2019) (**Figure 1**). *Poria cocos* grows underground on the colonization of Pinus species (Yang et al., 2019) and is extensively used in China as well as other East Asian countries for its various the rapeutic effects. Its clinical indications include promoting urination, removing dampness, invigorating the spleen, and calming the nerves (Kobira et al., 2012; Li et al., 2012; Wang et al., 2012a). Owning to its markedly medicinal function, few side effects and rich resources, the phytochemistry and pharmacology properties of *Poria cocos* have become a hot topic since the 1960s. Furthermore, previous studies have also showed that the chemical components of *Poria cocos* includ triterpenes, polysaccharides, and other minor components such as steroids, amino acids, and so on (Sun, 2014). According to the existing research literature, it can be clearly seen that the main active constituents of *Poria cocos* are concentrated on triterpenes and polysaccharides (Jia et al., 2016). Some of these constituents possessed a series of biological activities including anti-tumor (Sun and Xia, 2018), hepatoprotective (Wu et al., 2019), anti-inflammatory (Lee et al., 2017), anti-oxidant (Wang et al., 2016), anti-bacterial (Wang et al., 2019a), immunomodulation(Pu et al., 2019), etc. Therefore, these complex chemical compounds and pharmacological effects of *Poria cocos* attracted researcher’s considerable attention, meanwhile, they also brought huge challenges for research.

The objectives of the review are i) to summarize the chemical compounds and pharmacological effects of *Poria cocos*, ii) to update the latest published data about *Poria cocos*, and iii) to discuss some promising direction for further research on *Poria cocos*.

## Ethnobotanical Studies

*Poria cocos* is mainly native to East Asia and Southeast Asia and concentrated in regions with a subtropical and humid climate such as China, Vietnam, and Thailand. *Poria cocos* is a geographically representative product of Yunnan and Anhui in Hubei Province. Luotian County in Hubei Province was once approved the Good Agricultural Practice (GAP) planting demonstration base of *Poria cocos* (Rios, 2011).

Compatible with other Chinese medicines, *Poria cocos* can be usually formulated to ameliorate a variety of syndromes but is generally not used alone. First described in a classic prescription book, *Jin Kui Yao Lue* (***金匮要略***), Dang Gui Shao Yao San can be frequently applied in the treatment of anemia and ocular disorders and *Poria cocos* of this formula is used to eliminate damp and strengthen spleen (Shen et al., 2005). Another farmous formula, Gui Zhi Fu Ling Wan, which was also recorded in *Jin Kui Yao Lue* (***金匮要略***), can effectively promote blood circulation or removing stasis (Nozaki et al., 2014). In Gui Zhi Fu Ling Wan, *Poria cocos* has a similar effect such as resolving dampness and tonifying spleen as in Dang Gui Shao Yao San. Another classic formula containing *Poria cocos* is Wu Ling San, of which *Poria cocos* plays an irreplaceable role to clear out edemas induced by nephropathy, diabetes, and brain damage. Besides, there are other formulas containing *Poria cocos*, such as Si Jun Zi Tang recorded in the *Tai Ping Hui Min He Jue Fang* (***太平惠民和剂局方***), Zhu Ling Tang and Ling Gui Zhu Gan Tang both of which are documented in the *Shang Han Lun* (***伤寒论***) (Hsu et al., 1996). In the Chinese pharmacopoeia 2015 edition, the traditional Chinese medicine preparations containing *Poria cocos* accounted for nearly 15% (Zhu et al., 2018a). In conclusion, *Poria cocos* has generated irreplaceable effects in many prescriptions.

## Modern Quality Control

*Poria cocos* contains two main bioactive components, the triterpene acids and the polysaccharide fraction. Triterpenes, however, are generally regarded as the principal groups of chemicals of *Poria cocos* and often selected as the chemical markers to evaluate the quality of *Poria cocos* (Rios, 2011; Zhu et al., 2018b). Moreover, pachymic acid is specific to *Poria cocos* and do not exist in any other traditional Chinese medicine. In China, the quality *of Poria cocos* produced in Yunnan is the best. Many effective and credible methods including high performance liquid chromatography (HPLC), liquid chromatography (LC), liquid chromatography coupled with mass spectrometry (LC-MS), and DNA sequencing analysis to isolate and identify the active ingredients had been applied for the quality control of *Poria cocos* (Zhu et al., 2018a). Ultra-performance liquid chromatography-quadrupole/time- of-flight mass spectrometry (UHPLC-QTOF-MS/MS) was used to explore the differences of secondary metabolites in these three botanical parts (the epidermis, middle, and inner part) of Poria cocos. Fifteen chemical components which were common to all three parts, were unequivocally or tentatively identified and eight major bioactive triterpene acids were simultaneously quantified for quality evaluation (Zhu et al., 2018b). Ten compounds were screened out as potential markers to distinguish the quality of *Poria cocos* by UHPLC-QTOF-MS/MS (Zhu et al., 2018a).

## Constituents From *Poria cocos*

### Triterpenes

**Figure 2:**
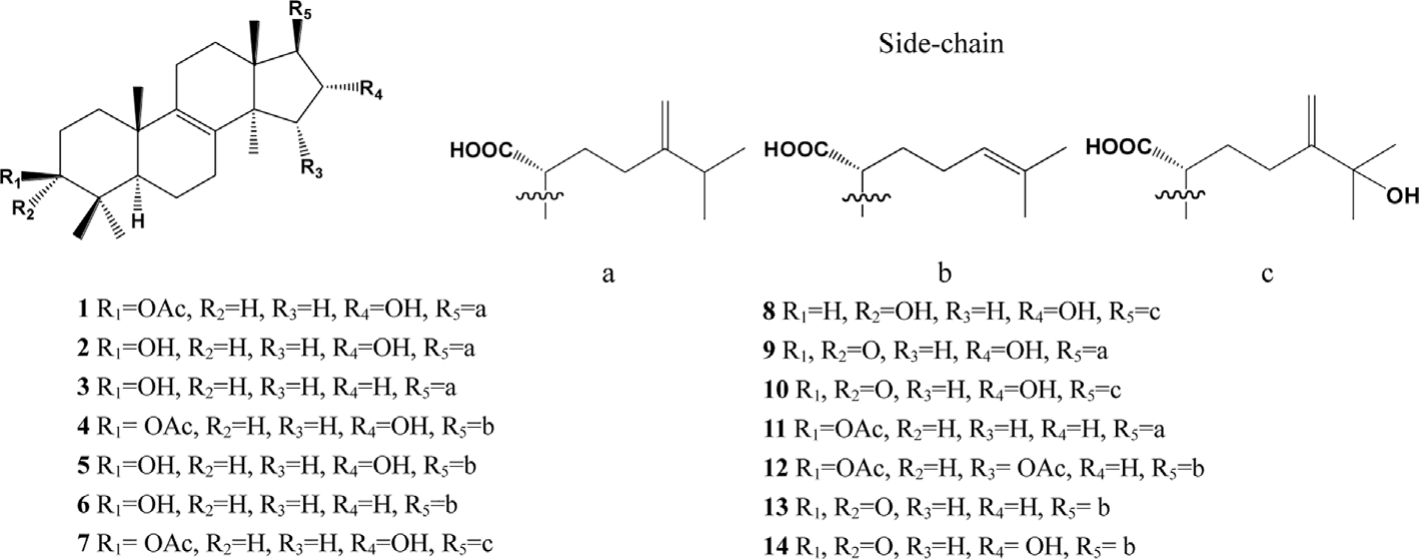
Structures of the lanosta-8-ene type triterpenes from *Poria cocos*.

**Figure 3:**
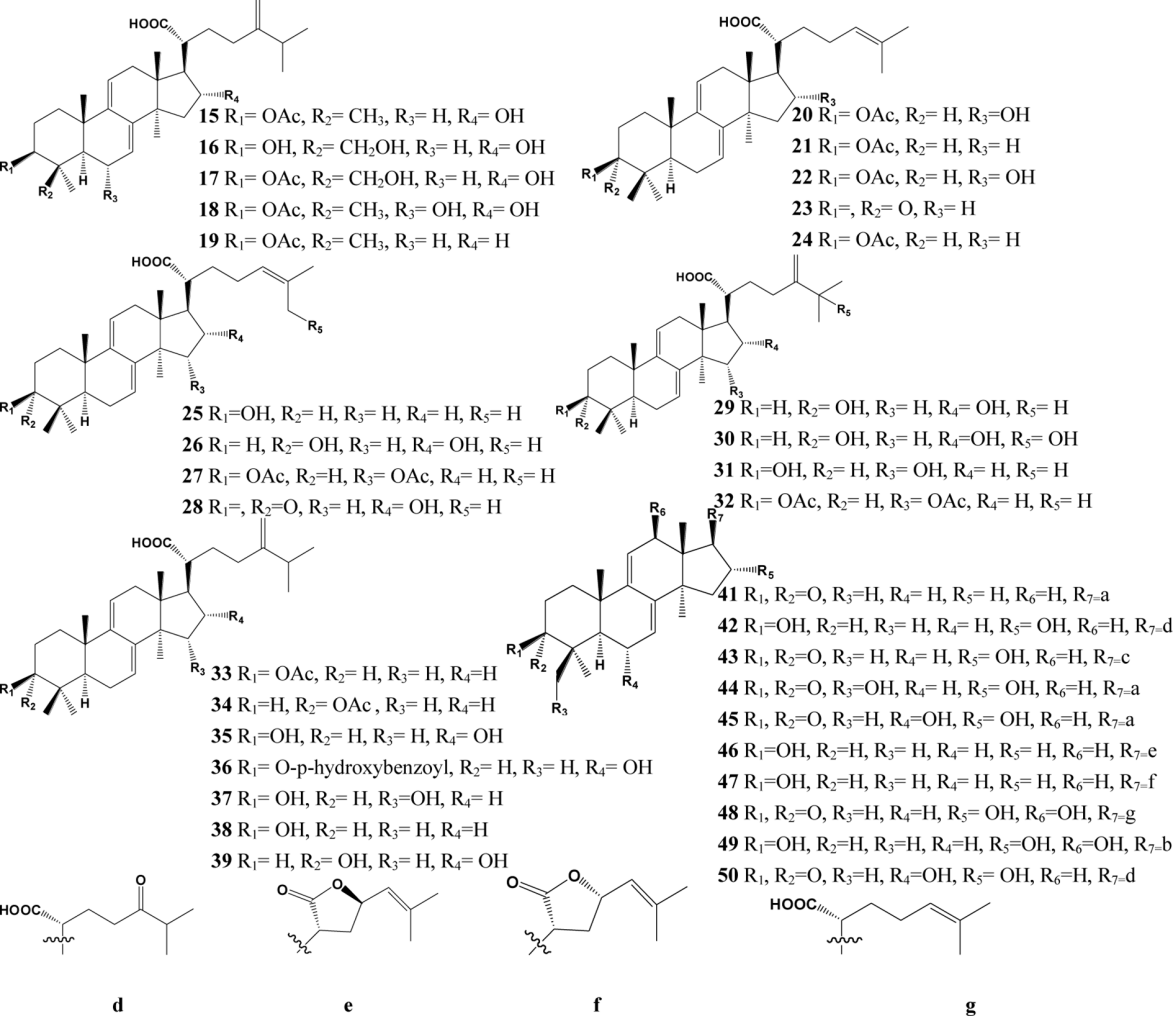
Structures of the Lanosta-7,9(11)-diene type triterpenes from *Poria cocos*.

**Figure 4:**
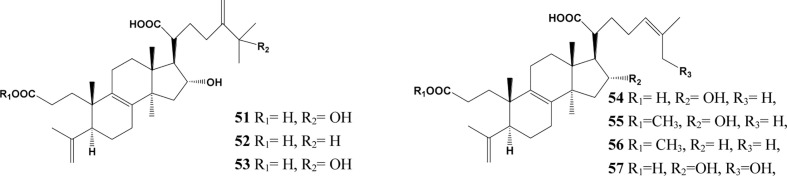
Structures of the 3,4-seco-lanostan-8-ene type triterpenes from *Poria cocos*.

**Figure 5:**
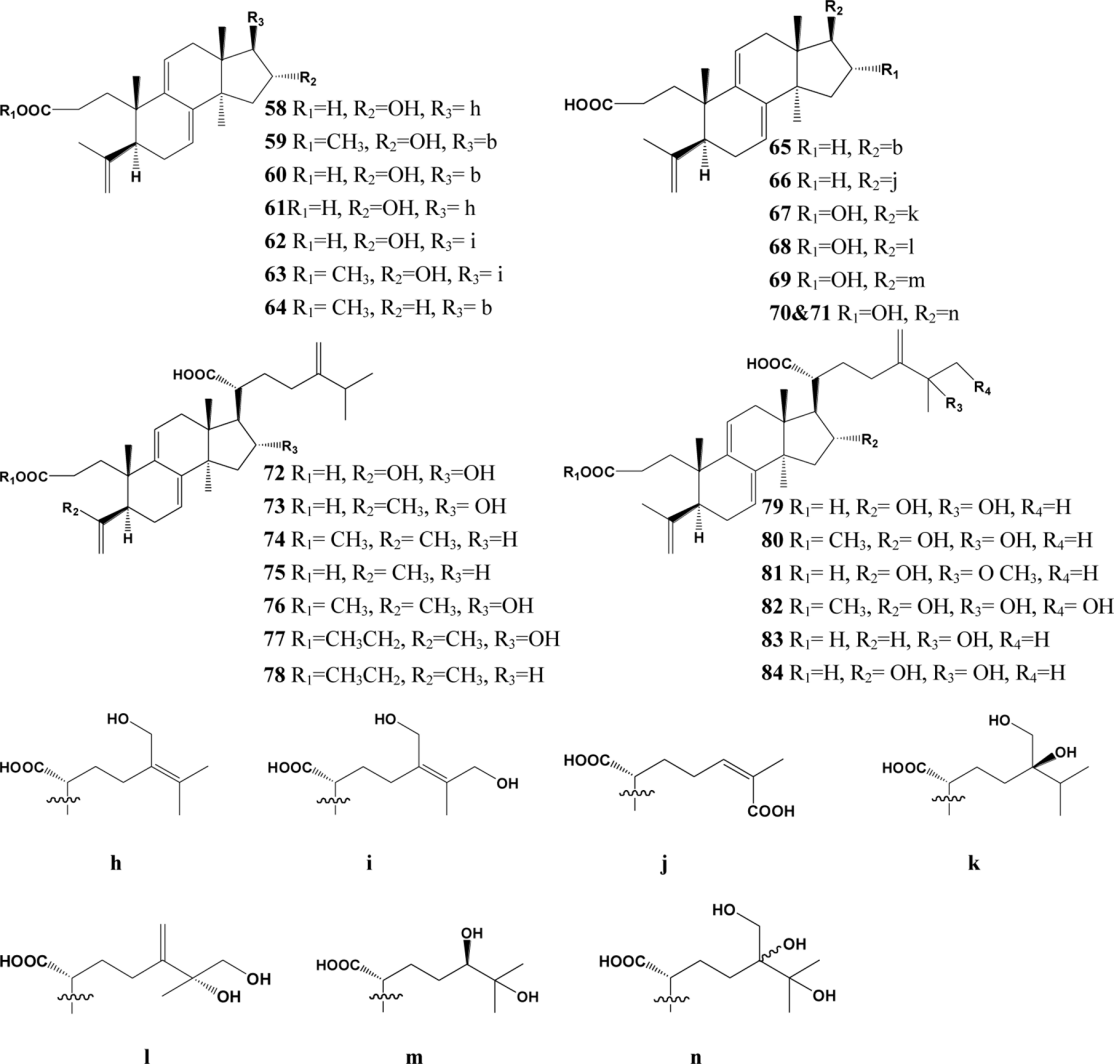
Structures of the 3,4-seco-lanostan-7,9(11)-diene type triterpenes from *Poria cocos*.

**Figure 6:**
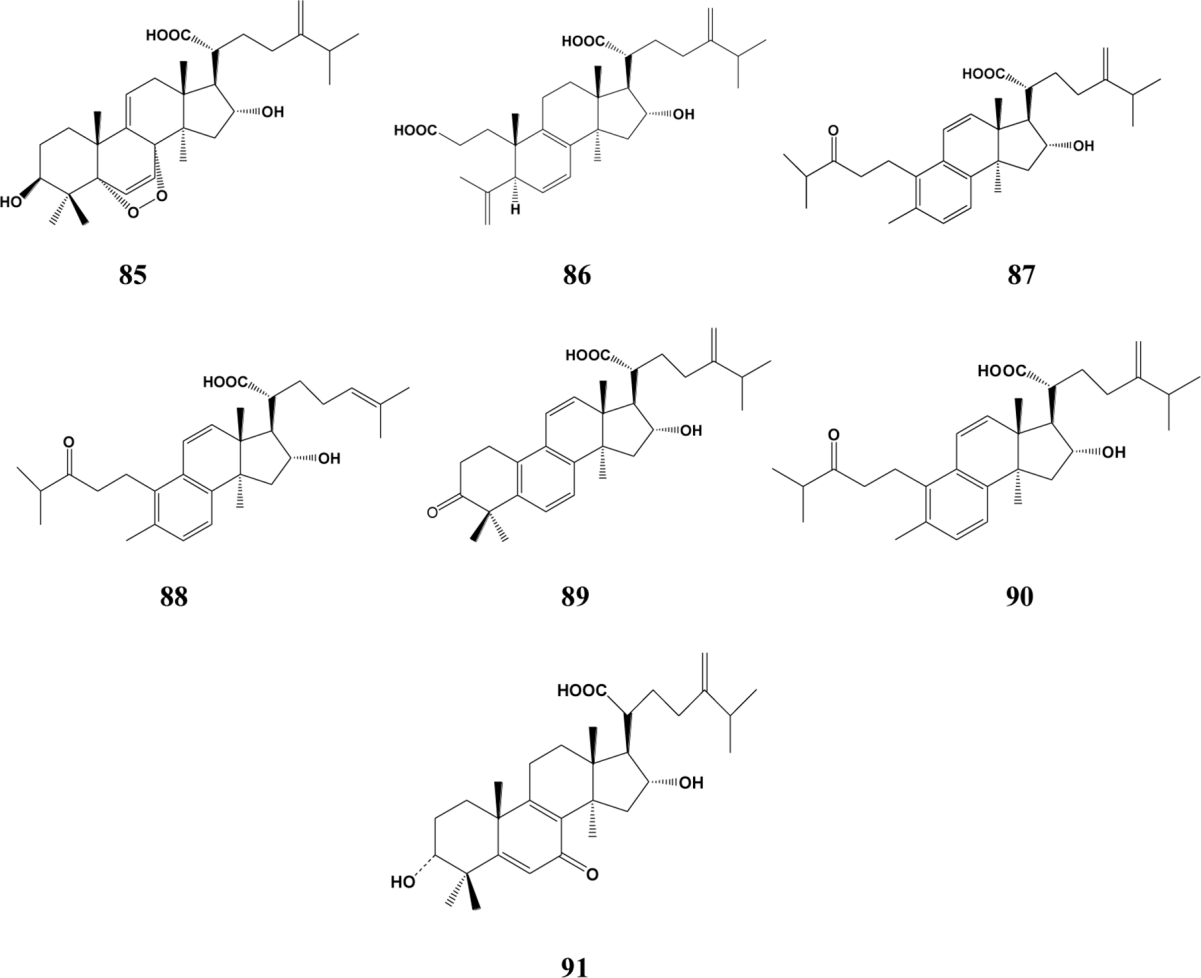
Structures of other type triterpenes from *Poria cocos*.

In the past decades, a total of 91 triterpenes, 1-91, were isolated and identified from *Poria cocos* and ascribed to derivations of lanostane or secolanostane skeletons (**Table 1** and **Figures 2**–**6**).

**Table 1:** Summary of the Lanosta-8-ene type triterpenes in *Poria cocos*.

S.N.	Origins	Compounds	Reference
Lanosta-8-ene type triterpenes			
1	Surface layer, Sclerotium	Pachymic acid	(Fu et al., 2018)
2	Sclerotium	Tumulosic acid	(Lv et al., 2013)
3	Surface layer	Eburicoic acid	(Huang et al., 2018)
4	Sclerotium	3-O-acetyl-16α-hydroxytrametenolic acid	(Wang et al., 2013)
5	Sclerotium	16α-Hydroxytrametenolic acid	(Wang et al., 2013)
6	Surface layer	Trametenolic acid	(Li et al., 2017)
7	Sclerotium	25-Hydroxypachymic acid	(Zheng and Yang, 2008a)
8	Surface layer	25-Hydroxy-3-epitumulosic acid	(Akihisa et al., 2009)
9	Surface layer	16α-Hydroxyeburiconic acid	(Wang et al., 2013)
10	Surface layer	16α, 25-Dihydroxyeburiconic acid	(Akihisa et al., 2009)
11	Surface layer	Eburicoic acid acetate	(Lee et al., 2018a)
12	Surface layer	Versisponic acid E	(Chen B. et al., 2019)
13	Surface layer	Pinicolic acid A	(Chen B. et al., 2019)
14	Surface layer	Pinicolic acid E	(Chen B. et al., 2019)
Lanosta-7,9(11)-diene type triterpenes			
15	Sclerotium	3β-Acetyloxy-16α-hydroxylanosta-7,9 (11),24(31)-trien-21-oic acid	(Shingu et al., 1992)
16	Sclerotium	29-Hydroxydehydrotumulosic acid	(Cai and Cai, 2011)
17	Sclerotium	29-Hydroxydehydropachymic acid	(Cai and Cai, 2011)
18	Sclerotium	16α-Hydroxydehydropachymic acid	(Nukaya et al., 1996)
19	Surface layer	Dehydroeburicoic acid monoacetate	(Lee et al., 2018b)
20	Sclerotium	3-O-acetyl-16α- hydroxydehydrotrametenolic acid	(Zeng et al., 2015)
21	Surface layer, Sclerotium	Dehydrotrametenolic acid	(Qian et al., 2018)
22	Sclerotium	3β,16α-Dihydroxylanosta-7,9(11),24- trien-21-oic acid	(Zeng et al., 2015)
23	Surface layer	Dehydrotrametenonic acid	(Akihisa et al., 2007)
24	Surface layer	3β-(Acetyloxy)lanosta-7,9(11),24-trien-21-oic acid	(Chen B. et al., 2019)
25	Surface layer, Sclerotium	3-epi-Dehydrotrametenolic acid	(Akihisa et al., 2007; Yang C. et al., 2010)
26	Surface layer	16α,27-Dihydroxydehydrotrametenoic acid	(Akihisa et al., 2009)
27	Surface layer	3,15-O-diacetyl-dehydrotrametenolic acid	(Chihara et al., 1970)
28	Surface layer	16α-Hydroxy-3-oxolanosta-7,9(11),24-trien-21-oic acid	(Chen B. et al., 2019)
29	Surface layer	3-epi-Dehydrotumulosic acid	(Wu et al., 2016a)
30	Surface layer, Sclerotium	25-Hydroxy-3-epidehydrotumulosic acid	(Ukiya et al., 2002)
31	Surface layer	Dehydrosulphurenic acid	(Peng et al., 2017)
32	Surface layer	Coriacoic acid B	(Chen B. et al., 2019)
33	Surface layer, Sclerotium	Dehydropachymic acid	(Jin et al., 2019)
34	Sclerotium	3-epi-Dehydropachymic acid	(Zhou et al., 2008)
35	Surface layer, Sclerotium	Dehydrotumulosic acid	(Ji et al., 2018)
36	Sclerotium	3β-p-Hydroxybenzoyl dehydrotumulosic acid	(Yasukawa et al., 1998)
37	Surface layer	15α-Hydroxydehydrotumulosic acid	(Zan et al., 2017)
38	Surface layer	Dehydroeburicoic acid	(Eom et al., 2018)
39	Surface layer	Poriacosone A	(Zheng and Yang, 2008a)
40	Sclerotium	Polyporenic acid C	(Cheng et al., 2019)
41	Surface layer	Dehydroeburiconic acid	(Lee et al., 2009)
42	Sclerotium	Poriacosone B	(Zheng and Yang, 2008a)
43	Surface layer	16α,25-Dihydroxydehydroeburiconic acid	(Akihisa et al., 2007)
44	Sclerotium	29-Hydroxypolyporenic acid C	(Cai and Cai, 2011)
45	Sclerotium	6α-Hydroxypolyporenic acid C	(Zheng and Yang, 2008b)
46	Surface layer	Porilactone A	(Chen B. et al., 2019)
47	Surface layer	Porilactone B	(Chen B. et al., 2019)
48	Surface layer	Pinicolic acid F	(Chen B. et al., 2019)
49	Surface layer	Poricoic acid ZL	(Chen L. et al., 2019)
50	Surface layer	Poricoic acid ZI	(Chen L. et al., 2019)
3,4-seco-lanostan-8-ene type triterpenes			
51	Surface layer	25-Hydroxyporicoic acid H	(Akihisa et al., 2007)
52	Surface layer	Poricoic acid H	(Ukiya et al., 2002)
53	Surface layer	Poricoic acid HM	(Akihisa et al., 2009)
54	Surface layer	Poricoic acid G	(Ukiya et al., 2002)
55	Surface layer	Poricoic acid GM	(Akihisa et al., 2009)
56	Surface layer	Poricoic acid ZK	(Chen B. et al., 2019)
57	Surface layer	Poricoic acid ZA	(Wang et al., 2017)
3,4-seco-lanostan-7,9(11)-diene type triterpenes			
58	Surface layer	Poricoic acid E	(Wang H. et al., 2015)
59	Surface layer	Poricoic acid BM	(Tai et al., 1995)
60	Surface layer, Sclerotium	Poricoic acid B	(Dong et al., 2015)
61	Surface layer	Poricoic acid I	(Chen B. et al., 2019)
62	Surface layer	Poricoic acid J	(Chen B. et al., 2019)
63	Surface layer	Poricoic acid JM	(Chen B. et al., 2019)
64	Surface layer	16-Deoxyporicoic acid BM	(Chen B. et al., 2019)
65	Surface layer, Sclerotium	16-Deoxyporicoic acid B	(Akihisa et al., 2007)
66	Sclerotium	3,4-seco-lanosta-4(28),7,9,24Z-tetraen- 3,26-dioic acid	(Yang L. et al., 2010)
67	Surface layer	Poricoic acid K	(Chen B. et al., 2019)
68	Surface layer	Poricoic acid L	(Chen B. et al., 2019)
69	Surface layer	Poricoic acid M	(Chen B. et al., 2019)
70	Surface layer	Poricoic acid N	(Chen B. et al., 2019)
71	Surface layer	Poricoic acid O	(Chen B. et al., 2019)
72	Surface layer	Poricoic acid F	(Tai et al., 1995)
73	Surface layer, Sclerotium	Poricoic acid A	(Qian et al., 2018)
74	Surface layer	Poricoic acid CM	(Akihisa et al., 2007)
75	Surface layer	Poricoic acid C	(Qian et al., 2018)
76	Surface layer	Poricoic acid AM	(Zhang W. et al., 2019)
77	Surface layer	Poricoic acid AE	(Yang C. et al., 2010)
78	Surface layer	Poricoic acid CE	(Yang C. et al., 2010)
79	Surface layer	Poricoic acid D	(Zhang G. et al., 2019)
80	Surface layer	Poricoic acid DM	(Akihisa et al., 2009)
81	Surface layer	25-Methoxyporicoic acid A	(Akihisa et al., 2009)
82	Surface layer	26-Hydroxyporicoic acid DM	(Akihisa et al., 2009)
83	Surface layer	25-Hydroxyporicoic acid C	(Akihisa et al., 2009)
84	Surface layer	Poricoic acid ZG	(Wang et al., 2018b)
Other type triterpenes			
85	Surface layer	5α,8α-Peroxydehydrotumulosic acid	(Akihisa et al., 2007)
86	Surface layer	6,7-Dehydroporicoic acid H	(Akihisa et al., 2009)
87	Surface layer	Daedaleanic acid D	(Chen B. et al., 2019)
88	Surface layer	Daedaleanic acid E	(Chen B. et al., 2019)
89	Surface layer	Daedaleanic acid F	(Chen B. et al., 2019)
90	Surface layer	Daedaleanic acid A	(Chen B. et al., 2019)
91	Surface layer	Poricoic acid ZH	(Wang et al., 2018b)

### Polysaccharides

**Figure 7:**
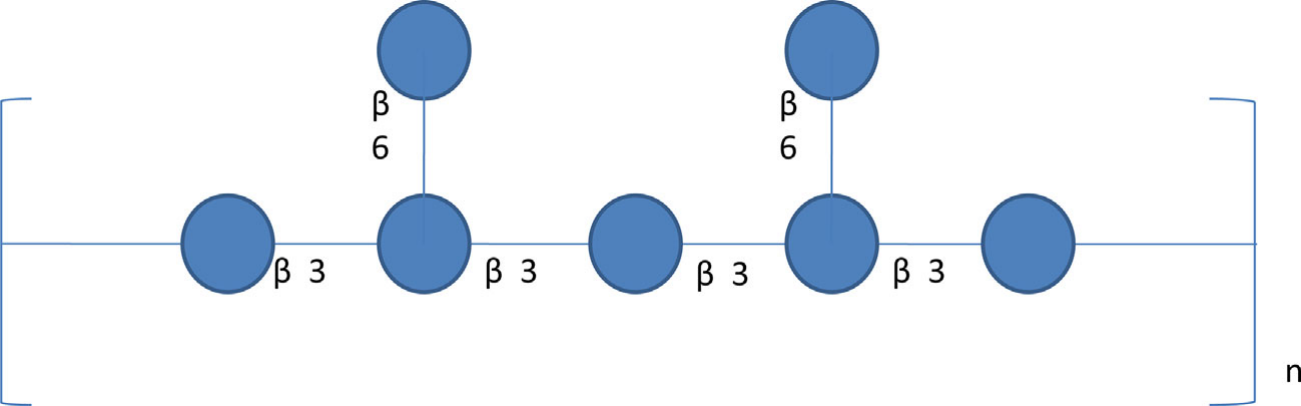
A schematic diagram of β-glucan structure in Poria cocos. β-Glucan is the major Poria cocos polysaccharide with β-(1→3) linked glucose backbone and β-(1→6)-linked glucose side chains. The β-glucan from Poria cocos has poor water solubility but decent anticancer activity.

*Poria cocos* polysaccharide (PCP) was extracted from the dried sclerotium of *Poria cocos* (84%, w/w), (Li X. et al., 2019) (**Table 2**). Different crude polysaccharide fractions were isolated by different solvent extraction methods, such as PCM1 (0.9% NaCl), PCM2 (hot water), PCSIII-2 (0.5 mol/L NaOH), PCM0 (MeOH), and PCP4-II (88% formic acid) (Wang et al., 2004a). Hence, PCP is undoubtedly a mixture of various types of polysaccharides, which consist of galactose, fucose, mannose, arabinose, xylose and glucose. β-glucan is regarded as the principal polysaccharide in *Poria cocos* with a (1→3)-linked glucose backbone main chain and some (1→6)-linked glucose side chains as shown in **Figure 7** (Jin et al., 2003a). To increase the water solubility of PCP, the side chains of the β-glucan was removed through the chemical reaction of periodate oxidation and smith degradation and the final product was named as “pachymaran” (Chihara et al., 1970). By carboxymethylation, the solubility and biological activity of PCP were further improved. Meanwhile, many chemical reactions including sulfation, carboxymethylation plus sulfation, methylation, hydroxpropylation, and hydroxyethylation have been also performed and these modified derivatives were also studied (Wang et al., 2004b; Huang and Zhang, 2005; Chen et al., 2010; Wang et al., 2012b). In general, these derivatives possessed better water-solubility performance and enhanced pharmacological activities.

**Table 2:** Summary of PCP from *Poria cocos*.

Compound name	Monosaccharide composition	Structural features	References
PCP	Manufactured by Hunan Butian Pharmaceutical Co, Ltd	ND	(Wu et al., 2019)
PCP	Manufactured by Hunan Butian Pharmaceutical Co, Ltd	ND	(Wu et al., 2018)
C-PCSG	ND	Carboxymethylated (1,3)-β-D-glucan	(Wang and Zhang, 2006; Wang et al., 2019a)
CMP33	ND	(1,3)-α-D-glucan with some (1,6)-α and (1,2)-α branches	(Liu et al., 2018)
PCP-1	Ara : Glu=0.02:1	Mw =2.33 kDa	(Tang et al., 2014)
PCP-2	Ara : Glu=0.01:1	Mw =3.20 kDa	(Tang et al., 2014)
PCP-3	Ara : Glu=0.03:1	Mw =2.85 kDa	(Tang et al., 2014)
CMP	Manufactured by Hunan Butian Pharmaceutical Co, Ltd	Carboxymethylated (1,3)-β-D-glucan with (1,2)-α branches	(Wang et al., 2018a; Wang et al., 2019a)
CMP33	ND	Mw = 15.23×10^4^ Da	(Liu et al., 2019)
PCP	Rib : Ara:Xyl : Man:Glu : Gal= 1.49:1.17:0.62:10.34:86.39:1.31	Mw = 160 kDa	(Pu et al., 2019)
PCP	Rib : Ara:Xyl : Man:Glu : Gal= 1.49:1.17:0.62:10.34:86.39:1.31	Mw = 160 kDa	(Tian et al., 2019)
PCWPS	Man : Glucose:Gal : Fuc= 30.073:16.599:41.470:10.103	Mw = 186209 Da	(Zhang et al., 2018)
PCWPW	Man : Glucose:Gal : Fuc= 36.896:7.298:40.480:15.326	Mw = 37154 Da	(Zhang et al., 2018)
PCP-II	Fuc : Man:Glu : Gal= 1.00:1.63:0.16:6.29	Mw = 29.0 kDa	(Lu et al., 2010; Wu L. et al., 2016)
S-P	ND	Sulfated (1,3)-α -D-glucan	(Wang W. et al., 2015)
CMP	ND	Carboxymethyl (1,3)-α-D-glucan	(Wang W. et al., 2015)
S-CMP	ND	Carboxymethylated-sulfated (1,3)-α-D-glucan	(Wang W. et al., 2015)
PCP-II	Fuc : Man:Glu : Gal= 1.00:1.63:0.16:6.29	Mw = 29.0 kDa	(Zhang G. et al., 2019)
PCP-II	Fuc : Man:Glu : Gal= 1.00:1.63:0.16:6.29	Mw = 29.0 kDa	(Wu Y. et al., 2016)
CMP1	ND	Mw = 25.27kDa	(Wang et al., 2012b)
CMP2	ND	Mw = 25.75kDa	(Wang et al., 2012b)
CMP3	ND	Mw = 27.88kDa	(Wang et al., 2012b)
CMP4	ND	Mw = 30.92kDa	(Wang et al., 2012b)
CMP5	ND	Mw = 36.00kDa	(Wang et al., 2012b)
PCP	Ara : Xyl:Man : Glc:Gal= 1.09:0.54:11.3:85.9:1.01	(1,3)-b-Glc, (1,4)- b-Man	(Ke et al., 2010)
H11	ND	(1,3)- (1,6)-β-D-glucan	(Kanayama et al., 1983)
PCP	Ara : Rib:Xyl : Man:Gal : Glu= 1.17: 1.49:0.62:10.34:1.31:86.39	ND	(Ke et al., 2010)
PCSC	Man : Gal:Ara=92:6.2:1.3	Mw = 8.0 kDa	(Lee et al., 2004)
Pi-PCM0	Ara : Xyl:Man : Gal:Glc= 2.5:1.5:70.6:18.5:7.0	Mw = 6.46 kDa	(Huang et al., 2007)
Pi-PCM1	Fuc : Ara:Xyl : Man:Gal : Glc= 10.9:1.0:2.8:23.6:36.5:25.2	Mw = 30.4 kDa	(Huang et al., 2007)
Pi-PCM2	Man : Gal:Glc=29.6:38.9:29.7	Mw = 103 kDa	(Huang et al., 2007)
PCM3-II	ND	(1,3) and (1,4) -β-D-glucan	(Zhang et al., 2006)
acPCM2	Fuc : Man:Gal : Glc=0.8:19.1:29.7:51.4	Mw = 17.0 kDa	(Jin et al., 2003b)
wcPCM2	Fuc : Man:Gal : Glc=3.4:12.5:13.4:70.7	Mw = 89.2 kDa	(Jin et al., 2003b)
CS-PCS3-II	carboxymethylated-sulfated derivative	(1→3)-β-D-glucan	(Chen et al., 2010)
ab-PCM3-I-S1	ND	Mw = 3.9 kDa	(Lin et al., 2004)
ab-PCM3-I-S2	ND	Mw = 11.3 kDa	(Lin et al., 2004)
ab-PCM3-I-S3	ND	Mw = 6.8 kDa	(Lin et al., 2004)
ab-PCM3-I-S4	ND	Mw = 5.8 kDa	(Lin et al., 2004)
ab-PCM3-I-S5	ND	Mw = 2.0 kDa	(Lin et al., 2004)
ac-PCM3-I-S1	ND	Mw = 17.4 kDa	(Lin et al., 2004)
ac-PCM3-I-S2	ND	Mw = 40.0 kDa	(Lin et al., 2004)
ac-PCM3-I-S3	ND	Mw = 26.1 kDa	(Lin et al., 2004)
ac-PCM3-I-S4	ND	Mw = 11.7 kDa	(Lin et al., 2004)
ac-PCM3-I-S5	ND	Mw = 4.7 kDa	(Lin et al., 2004)
S1	Sulfated (1,3)-α-D-glucan	Mw = 14.5 × 10^4^ Da	(Huang et al., 2006)
S2	Sulfated (1,3)-α-D-glucan	Mw = 9.10 × 10^4^ Da	(Huang et al., 2006)
S3	Sulfated (1,3)-α-D-glucan	Mw = 6.88 × 10^4^ Da	(Huang et al., 2006)
S4	Sulfated (1,3)-α-D-glucan	Mw = 4.71 × 10^4^ Da	(Huang et al., 2006)
S5	Sulfated (1,3)-α-D-glucan	Mw = 3.50 × 10^4^ Da	(Huang et al., 2006)
S6	Sulfated (1,3)-α-D-glucan	Mw = 2.65 × 10^4^ Da	(Huang et al., 2006)
WSP	ND	Mw = 1.75 × 10^5^ Da	(Bian et al., 2010)
WSP-1	ND	Mw = 1.86 × 10^6^ Da	(Bian et al., 2010)
WSP-2	ND	Mw = 3.58 × 10^4^ Da	(Bian et al., 2010)
PCP-H	Man : Gal:Glu: Ara= 0.92:0.18:86.88:12.01	ND	(Wang et al., 2016)
PCP-U	Man : Gal:Glu : Ara= 2.18:2.36:87.27:8.18	ND	(Wang et al., 2016)
PCP-E	Man : Gal:Glu : Ara= 1.98:0.36:81.72:15.93	ND	(Wang et al., 2016)
PCP-M	Man : Gal:Glu : Ara= 4.02:4.93:79.48:11.57	ND	(Wang et al., 2016)

Ara, araban; Xyl, xylose; Man, mannose; Glc, glucose; Gal, galactose; Fuc, fucose; Rha, rhamnose.

## Pharmacological Activities and Toxicological Information

Many experts and scholars have revealed that *Poria cocos* possessed remarkable pharmacological effects and complex mechanisms both *in vitro* and *in vivo*, as shown in **Table 3**.

**Table 3:** Summary of pharmacological activities and mechanisms of *Poria cocos*.

PharmacologicalEffects	Chemical component	Mechanism	Cell Lines/Model	Dosage of Administration	Ref.
Anticancer	H11	Inhibiting growth	subcutaneous mouse sarcoma S180	4 and 8 mg·kg^-1^	(Kanayama et al., 1986)
	Pi-PCM0, Pi-PCM1 and Pi-PCM2	inhibiting proliferation	Sarcoma 180 grown in mice	20 mg·kg^-1^	(Huang et al., 2007)
	PCM3-II	Reducing proliferation and viability and inducing cell-cycle G1 arrest	human breast carcinoma MCF-7 cells	400 μg·ml^-1^	(Zhang et al., 2006)
	ac-PCM2 and wc-PCM2	inhibiting growth	Sarcoma 180 solid tumor grown in BALB/c mice	20 mg·kg^-1^	(Jin et al., 2003b)
	CS-PCS3-II	increasing necrosis and apoptosis and immunological responses in tumor cells	Sarcoma 180 solid tumor grown in BALB/c mice	20 mg·kg^-1^	(Chen et al., 2010)
	S1- S6	inducing and facilitating apoptosis	HepG2 and S-180 tumor cells	20 mg·kg^-1^	(Huang et al., 2006)
	WSP, WSP-1 and WSP-2	anti-proliferation	S180 tumor cells	100 and 200 mg·kg^-1^	(Bian et al., 2010)
	CMP33	inhibiting growth	MCF-7, A549, HepG-2 and SGC-7901 cells	1 mg·ml-1	(Liu et al., 2019)
	Dehydropachymic acid and Dehydroeburicoic acid	anti-proliferative activity	Molt 4 and HL 60 cells	–	(Lai et al., 2016)
	Pachymic acid	inducing apoptosis by resulting in mitochondria dysfunction	DU145 cells	40 mg·kg-1	(Zhang et al., 2005)
	Pachymic acid	inhibiting-proliferation by activating caspase 3, up-regulating PTEN expression and reducing AKT phosphorylation	primary osteosarcoma cells	10–50 μg·ml^-1^	(Wen et al., 2018)
	Pachymic acid	Inhibiting proliferation and inducing apoptosis by up-regulating the expression of DNA damage-related proteins	NPC cells	10–30 μM	(Zhang et al., 2017)
	Pachymic acid	Decreased cell viability	SGC-7901 and MKN-49P cells	15–240 μmol·L^-1^	(Lu et al., 2017)
Anti-Oxidant	PCP-H, PCP-U, PCP-E and PCP-M	reducing and scavenging hydroxyl and DPPH radicals	–	–	(Wang et al., 2016)
	PCP-1, PCP-2 and PCP-3	scavenging hydroxyl radicals, ABTS radicals and ferrous ions	–	–	
Anti-inflammatory	PC-II	inhibiting the production of IP-10 induced by IFN-γ			(Lu et al., 2010)
	CMP33	improving colitis by decreasing levels of pro-inflammatory cytokines and increasing levels of anti-inflammatory cytokines	mice with inflammatory bowel disease (IBD)		(Liu et al., 2018)
	Pachymic acid, Trametenolic acid and Polyporenic acid C	inhibiting NO production and iNOS expression	RAW 264.7 cells		(Lee et al., 2017)
Immunomodulation	PCWPW and PCWPS	inhibited T cell proliferation	PC12 cells		(Zhang et al., 2018)
	S-P, CMP and S-CMP	Increasing hemolysin antibody titer and antibody	implanted HepG2 tumor in BALB/c mice		(Wang W. et al., 2015)
Kidney protection	Poricoic acid ZL, ZI and ZK	down-regulating profibrotic protein expression	HK-2, NRK-52E and NRK-49F cells		(Chen L. et al., 2019)
	Poricoic acid A	decreasing the elevated levels of creatinine and urea and improving renal fibrosis and podocyte injury	rats and renal NRK-52E cells		(Chen D. et al., 2019)
Liver protection	PCPs	decreasing the levels of ALT, LD, TNF-α and IL-6	liver injury mice induced by APAP		(Wu et al., 2018)

### Anti-Tumor Effects

**Figure 8:**
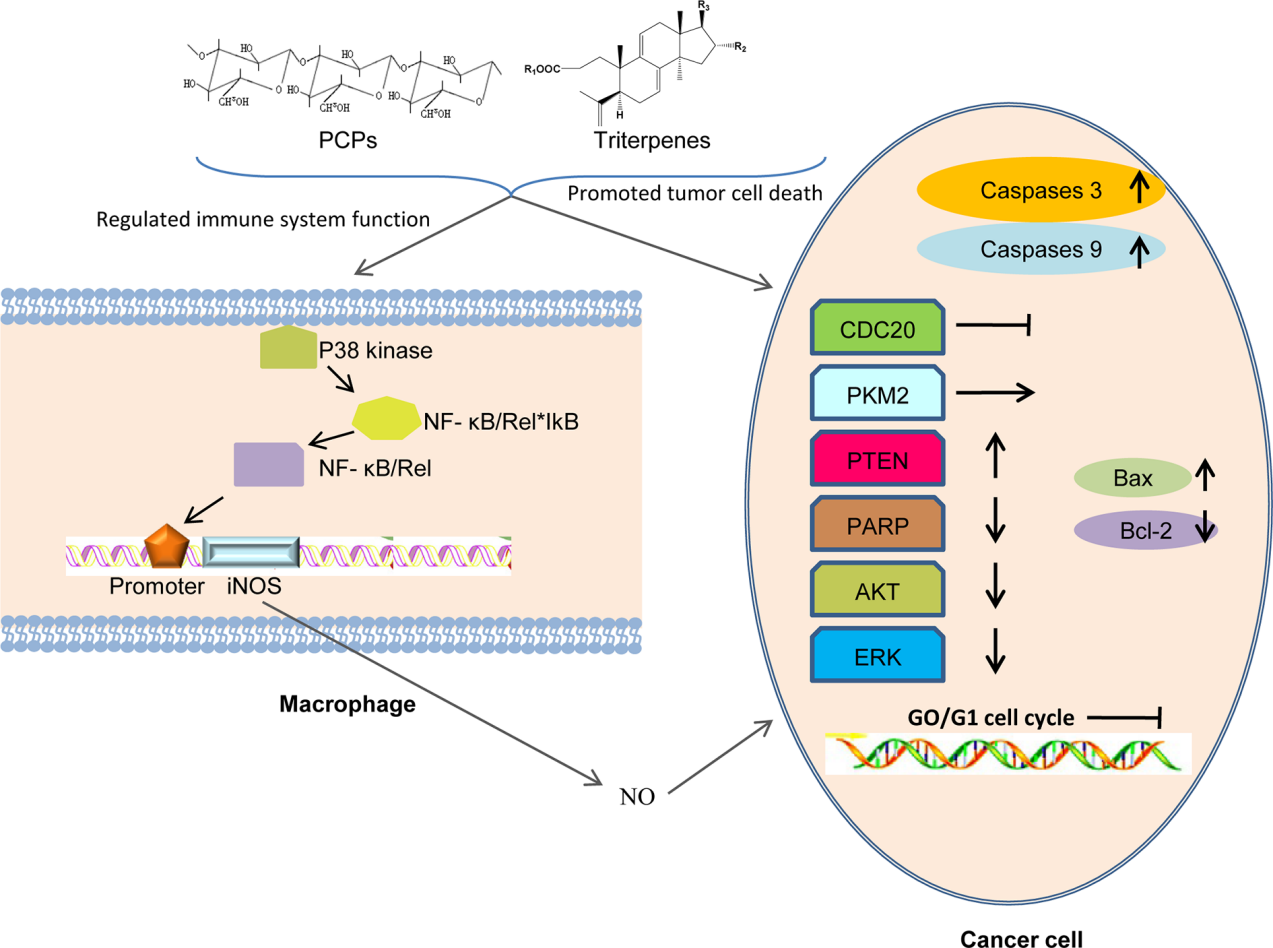
Possible anti-tumor mechanisms of PCPs. PCPs and triterpenes exert their antitumor activity *via* assisting the host to overcome adverse biological stresses, to enhance the lethality of macrophages by releasing cytokines to increase immunity, and to promote the apoptosis of tumor cells directly by up-regulating the expression of apoptosis-related genes.

One anticancer mechanism of PCP seemed to be related to the stimulation of cell-mediated immune responses (Lin and Zhang, 2004; Ke et al., 2010) (**Figure 8**). In 1983, a polysaccharide H11 with anti-cancer effects was firstly isolated from *Poria cocos*. Experiments demonstrated that H11 (4 and 8 mg·kg^-1^) had significant inhibition activity against subcutaneous mouse sarcoma S180 with inhibition ratio 94 and 96% respectively but no inhibition activity against ascites S180. H11 appeared to act through a host-mediated pathway rather than blocking tumor growth directly (Kanayama et al., 1983; Kanayama et al., 1986). Thirty and 100 μg·ml^-1^ PCSC, a PCP, could promote the production of NO (nitric oxide) and induce the transcription of iNOS (NO synthase) in RAW 264.7 macrophage cells by the activation of nuclear factor kB/Rel (NF-kB/Rel) pathway. Specifically, NF-kB/Rel pathway could be activated through strengthening the phosphorylation of IkB and p38 kinase. Moreover, NF-kB/Rel might translocate into the nucleus and bind to the promoter of iNOS gene (Lee et al., 2004). Three polysaccharides (Pi-PCM0, Pi-PCM1, and Pi-PCM2) derived from *Poria cocos*, all showed significant anti-proliferation effects on S-180 tumor-bearing BALB/c mice *in vivo* and on HL-60 tumor cell *in vitro* (Huang et al., 2007). After treating the breast cancer cells for 72 h with 12.5–400 μg·ml^-1^ of water-soluble β-glucan PCM3-II extracted from *Poria cocos*, the proliferation and viability of the MCF-7 cells was reduced dose-dependently and the cell-cycle G1 arrest was induced time-dependently. Mechanistically, the arrest was related with the down-regulations of unscheduled cyclin D1 and cyclin E expression. And increasing the ratio of Bax (pro-apoptosis)/Bcl-2 (anti-apoptosis) in breast cancer cells could induce apoptosis (Zhang et al., 2006).

Jin et al. found that the PCP cultured from wild strain in the medium containing corn steep liquor had the highest anti-tumor activity against S-180 *in vivo*, while the PCP cultured from cultivated strain in the medium containing bran extract had no obvious inhibitiory effects on tumor growth. studies on ac-PCM2 and wc-PCM2 showed that the higher molecular mass and better water solubility the polysaccharide possessed, the stronger the anti-tumor potency (Jin et al., 2003a). In BALB/c mice, the anti-tumor activity against S-180 of CS-PCS3-II, a derivative of PCS3-II, was markedly higher than that of PCS3-II. Histological examination showed that the S-180 tumor cells administrated with CS-PCS3-II appeared necrosis and even apoptosis, and the immunological responses in mice was enhanced (Chen et al., 2010). Compared with the native non-sulfated Pi-PCM3-I, sulfated derivatives (S1-S6) showed markedly higher anti-tumor activity against S-180 in mice and HepG2 and S-180 tumor cells, but lower toxicity was observed than 5-fluorouracil. The experiment results showed that S1-S6 time-dependently induced the apoptosis of HepG2 cell and facilitated the apoptosis of S-180 cells through regulating the expression of Bax and Bcl-2. It seemed that the sulfated derivative possessed the promise of drug exploitation as a chemotherapeutic drug (Huang et al., 2006; Liu et al., 2019). CMP is transformed into WSP by enzymic hydrolysis. WSP can be further separated to obtain WSP-1 and WSP-2. WSP-1, WSP-2, and WSP all exhibited strong anti-proliferation activity against S180 both *in vivo* as well as *in vitro*. Their inhibition rates *in vitro* were found to be 2.2 to 4.0%, higher than that of CMP. At the dose of 200 mg·kg^−1^, the inhibition rates of WSP, WSP-1 and WSP-2 *in vivo* were 43.94, 41.57, and 39.81%, respectively (Bian et al., 2010). CMP33, A carboxymethyl polysaccharide with triple-helix structure isolated from *Poria cocos*, exhibited a strong and dose-dependent inhibition efficiency on four cancer cells (MCF-7, A549, HepG-2 and SGC-7901) (Liu et al., 2019). After PCP sulfated, methylated, carboxymethylated, hydroxyethylated, and hydroxypropylated respectively, their anticancer activity were determined. The sulfated and carboxymethylated products had obvious anti-tumor effects on S-180, MKN-45 and SGC-7901 cells. Therefore, it might be concluded that good water solubility, relatively high chain stiffness and moderate molecular mass of the derivatives in aqueous solution seemed to increase the anti-tumor activity of polysaccharides (Wang et al., 2004b).

It was reported that compound 1 obviously inhibited cell multiplication and induced apoptosis of DU145 prostate cancer cells dose-dependently and time-dependently. Meanwhile, Compound 1 reduced bad phosphorylation, promoted the phosphorylation of Bcl-2 and activated caspases-3 and -9, indicating that it promoted apoptosis through inducing mitochondria dysfunction. Compound 1 also down-regulated the expression of proteins and decreased the activation of AKT signal pathway (Gapter et al., 2005). Compound 1 and 40 inhibited the inhibition activity against the expression of CDC20 which played an important role in cancer metastasis of PANC–1 cells dose–dependently by inhibiting the migration of pancreatic cancer cells (Cheng et al., 2019). Both compound 33 and 38 had a cytotoxicity effect on Molt 4 and HL 60 leukemic cell lines and targeting other than topoisomerases may be involved in the anti-proliferative activity (Lai et al., 2016). Compound 1 was discovered as a competing activator of PKM2, leading to a decreasing glucose uptake and lactate production in SK-BR-3 breast carcinoma cells, indicating that glycolysis was blocked or down-regulated to induce tumor cell proliferation (Miao et al., 2019). The results showed that compound **1** had markedly inhibition efficiency on human primary osteosarcoma cells proliferation concentration- and time-dependently. Meanwhile, compound 1 induced cell apoptosis dose-dependently, activated caspase 3, up-regulated PTEN expression and reduced AKT phosphorylation, demonstrating that compound 1 might be effective in treating human osteosarcoma (Wen et al., 2018). The anti-tumor activity of compound 1 was observed on nasopharyngeal carcinoma (NPC) cells and it was found that compound 1 might obviously inhibit cell proliferation and dose-dependently promote the apoptosis of the human NPC cells. Meanwhile, compound 1 caused morphological changes of the nucleus and up-regulated the expression of DNA damage-related proteins (Zhang et al., 2017). In addition, compound 1 could inhibit G0 phase arrest in gastric cancer cell lines SGC-7901 and MKN-49P. Moreover, Compound 1 regulated the expression of apoptosis-related proteins (caspase-3, PARP, Bcl-2, and Bax), suppressed the mitochondrial capacity of gastric cancer cells dose-dependently and finally induced cell apoptosis *in vitro*. Furthermore, compound 1 inhibited the tumor growth of xenograft models of gastric cancer and promoted the survival of animals obviously (Lu et al., 2017; Sun and Xia, 2018). In addition, compound 1 might inhibit tumorigenesis of gastric cancer cells through up-regulating the expression of Bax by suppressing hypoxia/HIF1α (Lu et al., 2018). Triterpene acids extracted from the epidermis of *Poria cocos* were observed to inhibit the growth of lung cancer cells A549 *in vitro* and *in vivo* and the IC50 value of compound 1, the most abundant chemical ingredients of the extract, was found to be 34.6 μg·ml^-1^, suggesting that compound 1 was the main anti-lung cancer ingredient in the triterpene acids (Dong et al., 2015). It was reported that compound 1 markedly inhibited the growth of gallbladder carcinoma cells dose- and time-dependently by inducing cell cycle arrest at G0 phase. Compound 1 also markedly reduced the migration and invasion of gallbladder carcinoma cells dose-dependently by suppressing cancer cell adhesion ability. Finally, it was demonstrated that compound 1 can inhibit gallbladder cancer tumorigenesis by affecting AKT and ERK signaling pathways (Chen et al., 2015).

### Anti-Oxidant Effects

Reactive oxygen species produced by normal metabolism, such as hydroxyl radicals (·OH), superoxide anions (·O^2-^), and hydrogen peroxides (H2O2) could induce the peroxidation of membrane lipids, thus causing various illnesses including cancer, aging and angiocardiopathy (Zhang et al., 2013).

PCPs were extracted from *Poria cocos* by hot water extraction (PCP-H), ultrasonic-assisted extraction (PCP-U), enzyme-assisted extraction (PCP-E), and microwave-assisted extraction (PCP-M), respectively. In vitro their antioxidant properties were determined on the basis of DPPH radical, reducing, power hydroxyl radical and metal chelating ability. PCP-M exhibited the highest reducing ability and strongest scavenging activity of hydroxyl radicals and DPPH radicals, while PCP-U showed the weakest antioxidant capacity (Wang et al., 2016). The water extracts from *Poria cocos* had protective effects on apoptosis in rat pheochro-mocytoma (PC12) cells apoptosis induced by Abeta1-42. The possible mechanisms were related to reducing the expression of Bax and the activity of caspase-3, indicating that *Poria cocos* had the potential to protect PC12 cells from apoptosis induced by oxidative stress (YH et al., 2009). Moreover, the water extracts showed the inhibition efficiency on lipid peroxidation induced by FeCl2-ascorbic acid in rat liver concentration-dependently. Its superoxide anion scavenging potency varied from 30.0 to 75.6%, and its anti-superoxide potency ranged from 38.5 to 81.4% with the concentrations from 0.1 to 10.0 mg·ml^-1^. It might be concluded that *Poria cocos* aqueous extracts exhibited a concentration-dependent anti-oxidant activity (Wu et al., 2004).

Compared with a native β-(1-3)-D-glucan obtained from *Poria cocos*, its carboxymethylated product had great improvement in solubility, ability to bind bile acids *in vitro*, and antioxidant activity. It can be hypothesized that the carboxymethylated derivative would have a beneficial effect on the decrease of cholesterol and blood pressure (Wang et al., 2009). Some researchers prepared the water-soluble oxidized product of (1-3)-β-D-glucan using TEMPO/NaBr/NaClO oxidation system with *Poria cocos* as raw material. The oxidation enhanced the bile acid binding *in vitro* by improving water solubility and structural changes of polysaccharides. In addition, the derivative also had hydroxyl radical scavenging activity *in vitro* (Wang et al., 2011). The antioxidant activities of polysaccharides PCP-1, PCP-2, and PCP-3 from the degradation of PCPs with different concentrations of H2O2 solution were studied by establishing *in vitro* systems, including scavenging effects on hydroxyl radicals, ABTS radicals and ferrous ions. The anti-oxidant properties of polysaccharides were concentration-dependent (Tang et al., 2014).

### Anti-Inflammatory Effects

It is well known that inflammatory reaction is a common pathological phenomenon and widely exists in a variety of diseases. Not only cancers are strongly linked to inflammatory reaction, but their staging and prognosis are inversely associated with the expression of genes related to inflammation (Kim et al., 2012; Ma et al., 2013). It was found that PC-II, a polysaccharide from *Poria cocos*, inhibited the IFN-γ-induced production of inflammation marker IP-10 dose-dependently, demonstrating that PC-II might be a promising lead compound in the development of novel anti-inflammatory agents (Lu et al., 2010). Notably, PC-II exhibited no toxicity to human vascular endothelial cells (ECs), indicating its safety. It was demonstrated that the expression of IP-10 was regulated by PC-II at the translational level rather than the transcriptional level, so it may participate in regulating inflammatory-related diseases (Lu et al., 2010). Lee et al. revealed that treatment with PCP obviously promoted NO production and iNOS transcription in mouse RAW 264.7 cells by activating NF-kB/Rel, indicating that PCP could induce macrophages to produce NO by inducing the iNOS gene expression (Lee and Jeon, 2003). The effects of CMP33 from *Poria cocos* on inflammatory bowel disease (IBD) were studied with colitis induced by TNBS in mice. It was observed that CMP33 obviously ameliorated the colitis in mice by decreasing the levels of pro-inflammatory cytokines and increasing the levels of anti-inflammatory cytokines in the serum and colon tissue of colitic mice, demonstrating that CMP33 could protect IBD in mice through the potential TPG (targeting protein group) and PMP (key protein-metabolite pathways) (Liu et al., 2018).

Six triterpenoids were isolated from *Poria cocos* and their effects on the levels of NO and PGE2 (prostaglandin E2) and on the expression of inducible iNOS and COX-2 (cyclooxygenase-2) in LPS-induced Raw 264.7 cells were observed. The results showed that compound 1, 4, 6, 24, and 40 might inhibit the production of NO and expression of iNOS in LPS-induced Raw 264.7 cells. And compound 1 decreased PGE2 level by down-regulating the expression of COX-2 (Lee et al., 2017). Compound 22 and 29 showed obvious inhibitory effects (IC50: 18.27 μM and 16.87 μM, respectively) on LPS-induced NO production by reducing the expression of inducible NO synthase enzymes in RAW 264.7 cells, which might be regulated *via* blocking the signaling pathway of activator protein-1 (Cai and Cai, 2011).

### Immunomodulation

The immunomodulatory activities and the potential mechanisms of PCPs in RAW 264.7 macrophages were explored. It was observed that the levels of nitric NO, TNF-α, IL-1β, IL-6, and calcium were increased by PCPs in RAW 264.7 macrophages and the immunomodulatory effects of PCPs might be associated with the Ca2+/PKC/p38/NF-κB signaling pathway (Pu et al., 2019). The levels of NO, IL-2, IL-6, IL-17 A, TNF, and IFN-γ were elevated in RAW 264.7 macrophages treated with PCPs and the expression of TLR4, MyD88, TRAF-6, p-NF-κB, and p-c-JUN was significantly enhenced in mice, demonstrating that PCPs might show immunomodulatory activity *via* TLR4/TRAF6/NF-κB signaling pathway (Tian et al., 2019). It was observed that PCWPW and PCWPS inhibited T cell proliferation induced by ConA dose-dependently, and PCWPS protected the PC12 cells from damage induced by H2O2 and inhibited B cell proliferation induced by LPS. These findings demonstrated that PCWPW and PCWPS have become promising immunosuppressive agents in food and pharmaceutical industries (Zhang et al., 2018). Furthermore, antigen-specific antibody levels in mice immunized with influenza vaccine were elevated by PCP-II, and the proliferation of splenocytes was improved. In addition, IL-12p70 and the production of TNF-α were induced by PCP-II. These results revealed that PCP-II-adjuvanted vaccines could strengthen humoral and cellular immunity (Wu et al., 2016). *Poria cocos* bark extract ameliorated the symptoms of food allergy (FA) and atopic dermatitis (AD) and increased the levels of Th2-related cytokines and the population of Foxp^3+^CD^4+^ Tregs in both AD and FA, revealing that PCB extract could be a novel oral immunosuppressive agents for treating AD and FA through the production of Tregs (Bae et al., 2016). PCPs *was* sulfated (S-P), carboxymethyl (CMP), and carboxymethylated-sulfated (S-CMP), respectively. Of the three derivatives, the S-CMP owned the best immunological activity *in vivo* and the highest inhibition ratio against the implanted HepG2 tumor in BALB/c mice, with notable rise of hemolysin antibody titer in serum, the increase of the production of spleen antibody and the delay of type hypersensitivity (Wang H. et al., 2015).

Compound 1, 2, 16, 17, 33, 35, 40, and 44 reduced the production of NO induced by LPS in RAW 264.7 cells dose-dependently. Of these, Compound 40 and 44 exhibited the higher inhibitory activity (IC50: 16.8 ± 2.7 μm and 18.2 ± 3.3μm, respectively). In addition, the inhibited NO release might be related to the intervention of protein-1 signaling pathway (Cai and Cai, 2011).

The results suggested that immunomodulatory protein from *Poria cocos* might upregulate TNF-α and IL-1β transcription and promote TNF-α production in RAW 264.7 cells (Li H. et al., 2019).

### Kidney Protection

It was observed that compound 49, 50, and 56 inhibited the expression of profibrotic protein in NRK-49F, NRK-52E, and HK-2 cells, indicating that the three kinds of poricoic acids could inhibit epithelial-mesenchymal transition (EMT). Compound 50 showed stronger inhibition of protein expression and activation of MMP-13 than compound 49 and 56. Thence, Compound 50 had potential as a novel agent for treating EMT and renal fibrosis (Chen L. et al., 2019). It was observed that compound 73 could weakened AKI-to-CKD transition in rats and renal NRK-52E cells. Firstly, compound 73 obviously decreased the elevated levels of creatinine and urea and improved renal fibrosis and cellular damage in IRI rats by inhibiting oxidative stress *via* NF-κB/Nrf2 pathways, indicating that compound 73 could block AKI-to-CKD transition through regulating growth arrest-specific 6 (Gas6)/Axl-NF -κB/Nrf2 signaling cascade (Chen D. et al., 2019). Wang et al. revealed that compound 84 and 91 isolated from *Poria cocos* attenuated renal injury *via* the Wnt/β-Catenin and TGF-β/Smad pathway and selectively attenuated the phosphorylation of Smad3 by blocking the interaction between SARA, TGFβI and Smad3 (Wang et al., 2018b). It was revealed that compound 57 had the capacity to inhibit RAS and further suppress TGFβ1/Smad pathway through inhibiting Smad2/3 phosphorylation *via* blocking Smad2/3-TGFβRI protein interaction, and compound 57 was implicated in activation of RAS/TGFβ1/Smad axis in HK-2 cells and podocytes, indicating that compound 57 played a beneficial role in renal fibrosis and podocyte injury and could be considered as a novel RAS inhibitor for treating CKD (Wang et al., 2017). The effect of *Poria cocos* hydroethanolic extract on nephrotic syndrome (NS) in rats were also evaluated. The results showed that the levels of urine protein and serum total protein (TP), albumin (Alb), globulin (Glo), total cholesterol (TC), and interlukin-4 (IL-4) were all improved in rats treated with PHE, indicating that PHE might be developed as a group of effective compounds for the treatment of NS (Zan et al., 2017). One previous study demonstrated that “Fu-Ling-Pi” treatment could improve CKD in major metabolic pathways including adenine metabolism and amino acid metabolism (Zhao et al., 2013).

### Hepatoprotective Effects

Wu et al. investigated the effects of PCPs on acetaminophen (APAP)-induced liver injury in mice. In mice treated with PCPs, the dropped ALT, LD, TNF-α and IL-6 serum level and the inhibited inflammatory infiltration and cell apoptosis in liver tissue were observed. The results indicated that PCPs had pharmacological activity against liver damage induced by APAP in mice, and the potential mechanisms were related to alleviating inflammatory reaction and apoptosis in liver cells (Wu et al., 2018). The reduced inflammatory cytokines (TNF-β and TNFsR-β), enzymological molecules (AST, ALT, and LDL), and heat shock protein 90 (Hsp90) levels were observed after APAP exposure, elucidating that PCPs had hepatoprotective effects on liver cells with the potential mechanisms of inhibiting cell death, reducing hepatocellular inflammatory stress and Hsp90 bioactivity (Wu et al., 2019).

### Anti-Bacterial Effects

The effects of CMP added with lotus seedpod oligomeric procyanidins (LSPC) on Escherichia coli 10899 were observed. When mixed with a small amount of LSPC, the antibacterial effect of CMP was synergistically enhanced, especially when the concentration of CMP was below its critical concentration (1.35 mg/ml) (Wang et al., 2019a). Antibacterial activity experiments demonstrated that the growth of the carboxymethylated derivative of PCPs significantly inhibited the growth of Pseudomonas aeruginosa (Wang et al., 2010).

### Others

CMP ameliorated the enteric dysbacteriosis induced by 5-FU through regulating the proportion of bacteroidetes, lactobacilli, and butyric acid-producing and acetic acid-producing bacteria as well as restoring the enteric flora diversity of CT26 tumor-bearing mice, which might be related to the intervention of the NF-κB, Nrf2-ARE and MAPK/P38 pathways (Wang C. et al., 2018). Research results showed that ethanol extract of cultured *Poria cocos* mycelia markedly increased urinary volume, Na^+^ and Cl^−^excretion, and Na^+^/K^+^ ratio, suggesting its obvious diuretic activity in rats (Hu et al., 2017). Experiments *in vitro* showed that 10, 20, and 40 μg/ml Trametenolic acid B protected SH-SY5Y cell against damage induced by OGD/R through inducing cellular proliferation and inhibiting LDH leakage. The results *in vivo* exhibited that TAB (20, 40, and 80 mg/kg) might obviously improve the neurological impairment score, encephaledema, neuronal cell loss and apoptosis, and inhibit brain infarction volume of the cerebral I/R injury rats. It manifested that TAB possessed neuroprotective potency against ODG/R and I/R damage by inhibiting miR-10a expression and activating PI3K/Akt/mTOR signaling pathway to reduce mitochondrial-mediated apoptosis, which provided a new insight for interpreting the underlying mechanisms of TAB’ neuroprotective effects and a candidate agent to treat cerebral I/R injury (Wang et al., 2019b). It was observed that the EtOH extract of *Poria cocos* sclerotia was able to inhibit MSC differentiation toward adipocytes and promote osteogenic differentiation of MSC (Lee et al., 2018a). In addition, PCP improved osteoclastogenesis induced by RANKL throug inhibiting NFATc1 activity and phosphorylation of ERK and STAT3 (Song et al., 2018).

### Toxicological Evidence

*Poria cocos* has low toxicity to mice and there was no problem with oral administration of 6–18 g per day (Cuellar et al., 1997). Xiao Banxia plus fuling decoction constituted of *Poria cocos*, *Pinellia ternata*, and *Zingiber officinale*, which was effective drug for vomiting. The mice were given Xiao Banxia plus fuling decoction at the maximum concentration (0.4 ml·10g^-1^ each mice for 2.23 g·ml^-1^, which was 382.29 times daily oral dose for adult in clinical) for three times within 24 h for 7 days. Then, the index of normal physiological state such as diet, stool and piss and death amount of the mice were observed and recorded. The results revealed that Xiao Banxia plus fuling decoction had no obvious toxic effect (Wang et al., 2013). Compound fuling and liquorice decoction contains *Poria cocos*, *Cinnamomum cassia*, *Prunus persica*, *Fritillariae Cirrhosae*, and *Anemarrhena asphodeloides*, which is usually used to treat chronic obstructive emphysema. Acute and long term toxicity test of compound fuling and liquorice decoction were executed. In the acute toxicity test, the rats were given compound fuling and liquorice decoction at the concentration of 720g·kg^-1^, which was 100 times patient’s daily administration dosage and all rats had no significant poisoning reaction. In long term toxicity test, there was no significant difference between the high-dose group (360 g·kg^-1^), the middle-dose group (180 g·kg^-1)^, the low-dose group (90 g·kg^-1)^ dose groups and the control group. Thus, we can draw conclusion that *Poria cocos* have no cumulative toxicity and is security for the clinical application (Wang et al., 2013).

## Conclusions

Over the years, *Poria cocos* has attracted increasing interest, and relevant phytochemical and pharmacological researches have validated its traditional uses. A lot of pharmacological effects, including anti-tumor, anti-oxidant, anti-inflammatory, hepatoprotective, antibacterial, kidney protection, and immunomodulation are summarized in the review. Furthermore, *Poria cocos* is secure for clinical application without obvious toxicity.

Pharmacological and phytochemical researches of the crude extracts and chemical composition isolated from *Poria cocos* are getting more and more researcher’s concerning recently. In 2006, PCPs-based product called “compound polysaccharide oral solution” was developed by Hunan Butian pharmaceutical company of China and was granted a Chinese patent (200610163425-X). The major ingredient in the patented product is CMP (95%, w/w). In 2015, “Polysaccharidum of *Poria cocos* oral solution” was approved by Chinese Food and Drug Administration with a certified drug number B20050015 for treating many kinds of cancers, hepatitis, and other diseases alone or as adjuvant drug during chemo- or radiation therapy for cancer patients (Li X. et al., 2019). The relationship between the molecular mass, chain stiffness and water solubility of PCPs and the anti-tumor activity needs to be further studied and confirmed. Besides, clinical trials of *Poria cocos* are still lacking, which limits its therapeutic application.

Due to the low yield, difficult separation, and purification of natural active polysaccharide from *Poria cocos*, its reports on the biological activity are mainly limited to the crude extract or derivative, and the fine structure of polysaccharides is unclear. The comprehensive application of biomodification and chemical modification may be a new direction to further elucidate the structure-activity relationship of PCPs and facilitate the development of new polysaccharide drugs or biomaterials.

In conclusion, PCPs and triterpenes are promising agents to treat various diseases or act as functional components in food products.

## Author Contributions

AN and YC searched the literature, collected the data, and drafted the manuscript. ZZ and CZ contributed to analysis and manuscript preparation. XZ and WJ helped in checking the chemical structures. AN and ZZ downloaded the documents and made classification. CZ and AN contributed comments for version of the manuscript. All authors contributed to the article and approved the submitted version.

## Funding

This work was supported by the Chinese Medical Association Clinical Pharmaceutical Branch Youth Fund (LCYX-Q025). The authors would like to thank Enago (www.enago.cn) for the English language review.

## Conflict of Interest

The authors declare that the research was conducted in the absence of any commercial or financial relationships that could be construed as a potential conflict of interest.
